# 1-Benzoyl-3-(4-*n*-butyl­phen­yl)thio­urea

**DOI:** 10.1107/S1600536811051774

**Published:** 2011-12-14

**Authors:** M. Khawar Rauf, Masahiro Ebihara, Amin Badshah, Imtiaz-ud-Din

**Affiliations:** a Department of Chemistry, Quaid-i-Azam University, Islamabad 45320, Pakistan; b Department of Chemistry, Faculty of Engineering, Gifu University Yanagido, Gifu 501-1193, Japan

## Abstract

The dihedral angle between the benzoyl and phenyl groups in the title compound, C_18_H_20_N_2_OS, is 30.57 (4)°. The crystal packing is characterized by N—H⋯O hydrogen bonds. In the crysta, pairs of N—H⋯S hydrogen bonds link the molecules into inversion dimers

## Related literature

For background to our work on the structural chemistry of *N*,*N*′-disubstituted thio­ures and for related structures, see: Khawer Rauf *et al.* (2009*a* ▶,*b* ▶). For bond-length data, see: Allen *et al.* (1987 ▶). For a description of the Cambridge Structural Database, see: Allen (2002 ▶).
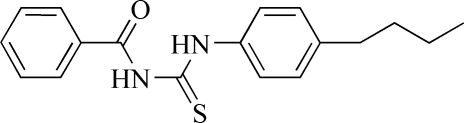


## Experimental

### 

#### Crystal data

C_18_H_20_N_2_OS
*M* *_r_* = 312.42
Triclinic, 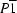

*a* = 4.648 (3) Å
*b* = 13.274 (8) Å
*c* = 13.690 (8) Å
α = 106.765 (7)°
β = 90.013 (6)°
γ = 92.700 (8)°
*V* = 807.9 (8) Å^3^
*Z* = 2
Mo *K*α radiation
μ = 0.20 mm^−1^
*T* = 123 K
0.40 × 0.10 × 0.10 mm

#### Data collection

Rigaku/MSC Mercury CCD diffractometer
6380 measured reflections
3632 independent reflections
3242 reflections with *I* > 2σ(*I*)
*R*_int_ = 0.043

#### Refinement

*R*[*F* ^2^ > 2σ(*F* ^2^)] = 0.043
*wR*(*F* ^2^) = 0.103
*S* = 1.06
3632 reflections
200 parameters
H-atom parameters constrained
Δρ_max_ = 0.33 e Å^−3^
Δρ_min_ = −0.27 e Å^−3^

### 

Data collection: *CrystalClear* (Molecular Structure Corporation & Rigaku, 2001 ▶); cell refinement: *CrystalClear*; data reduction: *CrystalClear*; program(s) used to solve structure: *SIR97* (Altomare *et al.*, 1999 ▶); program(s) used to refine structure: *SHELXL97* (Sheldrick, 2008 ▶); molecular graphics: *ORTEPII* (Johnson, 1976 ▶) and *TEXSAN* (Molecular Structure Corporation & Rigaku, 2004 ▶); software used to prepare material for publication: *Yadokari-XG 2009* (Kabuto *et al.*, 2009 ▶).

## Supplementary Material

Crystal structure: contains datablock(s) I, global. DOI: 10.1107/S1600536811051774/hg5142sup1.cif

Structure factors: contains datablock(s) I. DOI: 10.1107/S1600536811051774/hg5142Isup2.hkl

Supplementary material file. DOI: 10.1107/S1600536811051774/hg5142Isup3.cml

Additional supplementary materials:  crystallographic information; 3D view; checkCIF report

## Figures and Tables

**Table 1:** Hydrogen-bond geometry (Å, °)

*D*—H⋯*A*	*D*—H	H⋯*A*	*D*⋯*A*	*D*—H⋯*A*
N1—H1⋯O1	0.88	1.88	2.630 (2)	142
N2—H2⋯S1^i^	0.88	2.76	3.550 (2)	151

Symmetry code: (i) 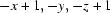.

**Fig. 1.**
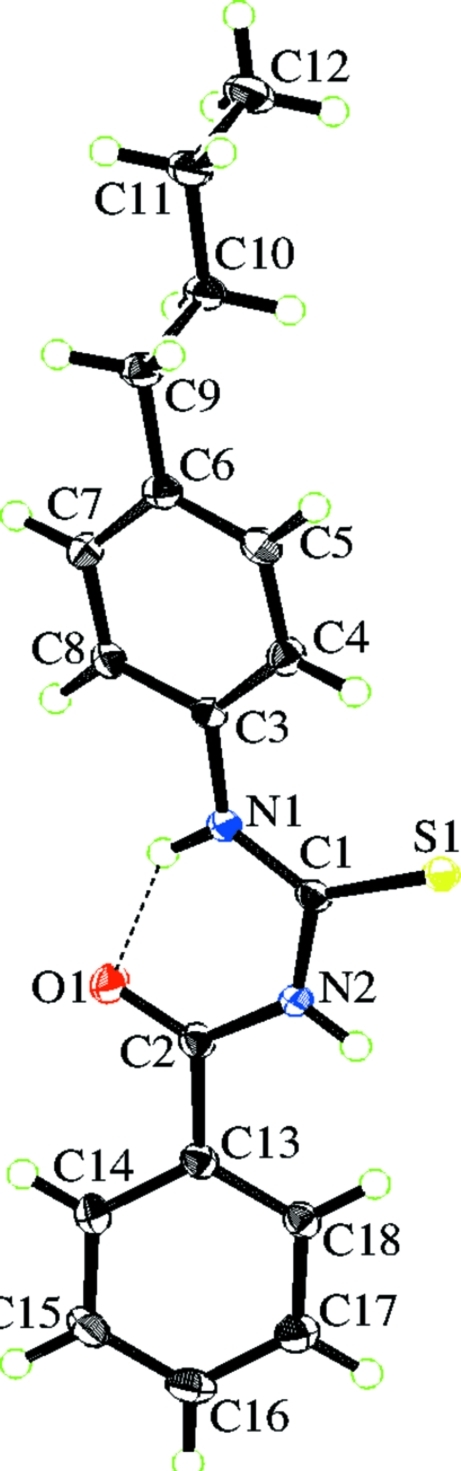
ORTEP of (I). Displacement ellipsoids are drawn at the 50% probability level. Hydrogen bonds showns as dashed lines.

**Fig. 2.**
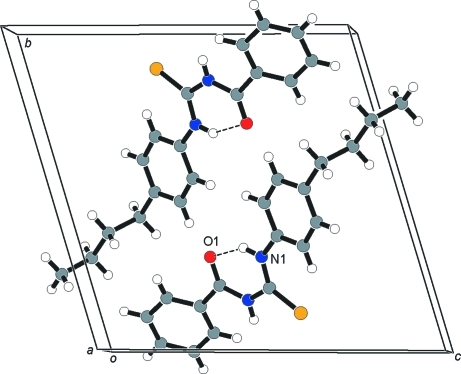
Packing diagram of (I). Hydrogen bonds shown as dashed lines.

## supplementary crystallographic information

### Comment

The background to this study has been set out in our previous work for the structural chemistry of *N*,*N'*-disubstituted thiourea (Khawar Rauf *et al.*, 2009*a*, 2009*b*). Herein, as a continuation of these crytallographic studies, the structure of the title compound (I) is described, Fig. 1. Compared to *N*-benzoyl-*N'*-phenylthioureas [Cambridge Structural Database (*Mogul* Version 1.7; Allen, 2002)and (Allen *et al.*, 1987)], the *n*-butyl substitution at C(6) on phenyl ring, implies no significant effect on these bond lengths. and show the molecule to exist in the thione form with typical thiourea C—S and C—O bonds, as well as shortened C—N bond lengths. The dihedral angles to the N(1) C(1)S(1) N(2) C(2)O(1) plane are 23.66 (8)° for the ring formed by C(13) to C(18) and 8.16 (9)° for the ring formed by C(3) to C(8). An intramolecular N—H···O H–bond is present (Table 1), forming a six-membered ring commonly observed in this class of compounds (Khawar Rauf *et al.*, 2009*a*, 2009*b*). In the crystal packing of (I), intermolecular N—H···S H–bonds link the molecules into centrosymmetric dimers (Fig.2).

### Experimental

Freshly prepared benzoylisothiocyanate (1.63 g, 10 mmol) was dissolved in acetone (30 ml) and stirred for 30 minutes. Afterwards neat 4-*n*-butylaniline (1.49 g, 10 mmol) was added and the resulting mixture was stirred for 2 h. The reaction mixture was then poured into acidified water and stirred well. The solid product was separated and washed with deionized water and purified by recrystallization from methanol/ 1,1-dichloromethane (1:1 *v/v*) to give fine crystals of the title compound (I), with an overall yield of 95%.

### Refinement

Some strong reflections were saturated in the experimental condition and excluded from the refinement. Hydrogen atoms were included in calculated positions and refined as riding on their parent atom with N—H = 0.88 Å and *U*_iso_(H) = 1.2U(N_eq_), C_aromatic_—H = 0.95 Å and *U*_iso_(H) = 1.2U(C_eq_) or C—H = 0.98–0.99 Å and *U*_iso_(H) = 1.5U(C_eq_), for butyl C atoms.

### Crystal data

**Table:**

C_18_H_20_N_2_OS	*Z* = 2
*M**_r_* = 312.42	*F*(000) = 332
Triclinic, *P*1	*D*_x_ = 1.284 Mg m^−^^3^
Hall symbol: -P 1	Mo *K*α radiation, λ = 0.71070 Å
*a* = 4.648 (3) Å	Cell parameters from 2558 reflections
*b* = 13.274 (8) Å	θ = 3.1–27.5°
*c* = 13.690 (8) Å	µ = 0.20 mm^−^^1^
α = 106.765 (7)°	*T* = 123 K
β = 90.013 (6)°	Needle like, colorless
γ = 92.700 (8)°	0.40 × 0.10 × 0.10 mm
*V* = 807.9 (8) Å^3^	

### Data collection

**Table:**

Rigaku/MSC Mercury CCD diffractometer	3242 reflections with *I* > 2σ(*I*)
Radiation source: Rotating Anode	*R*_int_ = 0.043
Graphite Monochromator	θ_max_ = 27.5°, θ_min_ = 3.1°
Detector resolution: 14.6199 pixels mm^-1^	*h* = −6→3
dtintegrate.ref scans	*k* = −17→14
6380 measured reflections	*l* = −13→17
3632 independent reflections	

### Refinement

**Table:**

Refinement on *F*^2^	Primary atom site location: structure-invariant direct methods
Least-squares matrix: full	Secondary atom site location: difference Fourier map
*R*[*F*^2^ > 2σ(*F*^2^)] = 0.043	Hydrogen site location: inferred from neighbouring sites
*wR*(*F*^2^) = 0.103	H-atom parameters constrained
*S* = 1.06	*w* = 1/[σ^2^(*F*_o_^2^) + (0.0399*P*)^2^ + 0.4308*P*] where *P* = (*F*_o_^2^ + 2*F*_c_^2^)/3
3632 reflections	(Δ/σ)_max_ < 0.001
200 parameters	Δρ_max_ = 0.33 e Å^−^^3^
0 restraints	Δρ_min_ = −0.27 e Å^−^^3^

### Special details

**Table:**

Geometry. All esds (except the esd in the dihedral angle between two l.s. planes) are estimated using the full covariance matrix. The cell esds are taken into account individually in the estimation of esds in distances, angles and torsion angles; correlations between esds in cell parameters are only used when they are defined by crystal symmetry. An approximate (isotropic) treatment of cell esds is used for estimating esds involving l.s. planes.
Refinement. Refinement of *F*^2^ against ALL reflections. The weighted *R*-factor *wR* and goodness of fit *S* are based on *F*^2^, conventional *R*-factors *R* are based on *F*, with *F* set to zero for negative *F*^2^. The threshold expression of *F*^2^ > σ(*F*^2^) is used only for calculating *R*-factors(gt) *etc*. and is not relevant to the choice of reflections for refinement. *R*-factors based on *F*^2^ are statistically about twice as large as those based on *F*, and *R*- factors based on ALL data will be even larger.

### Fractional atomic coordinates and isotropic or equivalent isotropic displacement parameters (Å^2^)

**Table:**

	*x*	*y*	*z*	*U*_iso_*/*U*_eq_	
C1	0.4183 (3)	0.19623 (11)	0.53711 (10)	0.0172 (3)	
S1	0.33263 (9)	0.11726 (3)	0.60881 (3)	0.02424 (12)	
N1	0.3191 (3)	0.29075 (10)	0.54395 (9)	0.0180 (3)	
H1	0.3862	0.3194	0.4975	0.022*	
N2	0.6153 (3)	0.16024 (10)	0.45859 (9)	0.0178 (3)	
H2	0.6907	0.0999	0.4556	0.021*	
C2	0.7060 (3)	0.20705 (12)	0.38572 (11)	0.0190 (3)	
O1	0.6324 (3)	0.29449 (9)	0.38503 (9)	0.0271 (3)	
C3	0.1241 (3)	0.35335 (11)	0.61301 (11)	0.0165 (3)	
C4	−0.0402 (3)	0.32010 (12)	0.68386 (12)	0.0212 (3)	
H4	−0.0285	0.2506	0.6890	0.025*	
C5	−0.2219 (3)	0.39021 (13)	0.74715 (12)	0.0217 (3)	
H5	−0.3322	0.3674	0.7959	0.026*	
C6	−0.2477 (3)	0.49186 (12)	0.74157 (11)	0.0190 (3)	
C7	−0.0851 (3)	0.52320 (12)	0.66869 (11)	0.0210 (3)	
H7	−0.1008	0.5922	0.6624	0.025*	
C8	0.0986 (3)	0.45522 (12)	0.60552 (11)	0.0201 (3)	
H8	0.2083	0.4781	0.5566	0.024*	
C9	−0.4331 (3)	0.56761 (13)	0.81559 (11)	0.0227 (3)	
H9B	−0.6086	0.5291	0.8295	0.027*	
H9A	−0.4937	0.6221	0.7845	0.027*	
C10	−0.2717 (3)	0.62093 (13)	0.91620 (11)	0.0218 (3)	
H10B	−0.2076	0.5659	0.9459	0.026*	
H10A	−0.0976	0.6598	0.9017	0.026*	
C11	−0.4509 (4)	0.69711 (14)	0.99458 (12)	0.0264 (3)	
H11B	−0.5213	0.7509	0.9644	0.032*	
H11A	−0.6208	0.6580	1.0117	0.032*	
C12	−0.2805 (4)	0.75183 (15)	1.09179 (13)	0.0329 (4)	
H12C	−0.2089	0.6989	1.1217	0.049*	
H12A	−0.4055	0.7983	1.1406	0.049*	
H12B	−0.1174	0.7935	1.0757	0.049*	
C13	0.8938 (3)	0.14395 (12)	0.30440 (11)	0.0185 (3)	
C14	0.9027 (3)	0.16959 (13)	0.21251 (12)	0.0247 (3)	
H14	0.7984	0.2268	0.2049	0.030*	
C15	1.0633 (4)	0.11182 (14)	0.13232 (12)	0.0281 (4)	
H15	1.0667	0.1287	0.0695	0.034*	
C16	1.2191 (4)	0.02936 (14)	0.14376 (12)	0.0275 (4)	
H16	1.3290	−0.0102	0.0887	0.033*	
C17	1.2152 (3)	0.00445 (13)	0.23512 (12)	0.0245 (3)	
H17	1.3228	−0.0520	0.2428	0.029*	
C18	1.0537 (3)	0.06196 (12)	0.31573 (11)	0.0190 (3)	
H18	1.0526	0.0452	0.3787	0.023*	

### Atomic displacement parameters (Å^2^)

**Table:**

	*U*^11^	*U*^22^	*U*^33^	*U*^12^	*U*^13^	*U*^23^
C1	0.0203 (7)	0.0183 (7)	0.0122 (6)	0.0008 (5)	0.0007 (5)	0.0033 (5)
S1	0.0384 (2)	0.01744 (19)	0.0190 (2)	0.00574 (15)	0.01041 (15)	0.00803 (14)
N1	0.0224 (6)	0.0170 (6)	0.0155 (6)	0.0029 (5)	0.0043 (5)	0.0061 (5)
N2	0.0225 (6)	0.0157 (6)	0.0157 (6)	0.0036 (5)	0.0035 (5)	0.0050 (5)
C2	0.0219 (7)	0.0185 (7)	0.0172 (7)	0.0005 (5)	0.0018 (5)	0.0062 (6)
O1	0.0365 (6)	0.0215 (6)	0.0274 (6)	0.0072 (5)	0.0122 (5)	0.0130 (5)
C3	0.0179 (7)	0.0171 (7)	0.0136 (6)	0.0014 (5)	0.0001 (5)	0.0028 (5)
C4	0.0213 (7)	0.0205 (7)	0.0227 (7)	0.0013 (6)	0.0026 (6)	0.0078 (6)
C5	0.0209 (7)	0.0258 (8)	0.0189 (7)	0.0008 (6)	0.0038 (6)	0.0073 (6)
C6	0.0165 (7)	0.0227 (8)	0.0157 (7)	0.0019 (5)	−0.0034 (5)	0.0019 (6)
C7	0.0256 (8)	0.0178 (7)	0.0196 (7)	0.0039 (6)	−0.0004 (6)	0.0052 (6)
C8	0.0242 (7)	0.0214 (7)	0.0163 (7)	0.0016 (6)	0.0021 (5)	0.0078 (6)
C9	0.0202 (7)	0.0267 (8)	0.0188 (7)	0.0056 (6)	−0.0002 (6)	0.0020 (6)
C10	0.0198 (7)	0.0264 (8)	0.0172 (7)	0.0041 (6)	0.0012 (6)	0.0029 (6)
C11	0.0261 (8)	0.0318 (9)	0.0186 (8)	0.0060 (7)	0.0029 (6)	0.0023 (7)
C12	0.0399 (10)	0.0363 (10)	0.0180 (8)	0.0041 (8)	0.0022 (7)	0.0004 (7)
C13	0.0203 (7)	0.0189 (7)	0.0163 (7)	−0.0016 (5)	0.0020 (5)	0.0055 (6)
C14	0.0300 (8)	0.0260 (8)	0.0214 (8)	0.0048 (6)	0.0040 (6)	0.0116 (6)
C15	0.0361 (9)	0.0335 (9)	0.0161 (7)	0.0021 (7)	0.0052 (6)	0.0091 (7)
C16	0.0299 (8)	0.0286 (9)	0.0208 (8)	0.0022 (7)	0.0077 (6)	0.0020 (7)
C17	0.0241 (8)	0.0237 (8)	0.0250 (8)	0.0026 (6)	0.0032 (6)	0.0058 (6)
C18	0.0203 (7)	0.0203 (7)	0.0165 (7)	−0.0014 (6)	0.0005 (5)	0.0058 (6)

### Geometric parameters (Å, °)

**Table:**

C1—N1	1.335 (2)	C9—H9A	0.9900
C1—N2	1.4028 (19)	C10—C11	1.523 (2)
C1—S1	1.6659 (16)	C10—H10B	0.9900
N1—C3	1.4228 (18)	C10—H10A	0.9900
N1—H1	0.8800	C11—C12	1.521 (2)
N2—C2	1.3755 (19)	C11—H11B	0.9900
N2—H2	0.8800	C11—H11A	0.9900
C2—O1	1.2282 (19)	C12—H12C	0.9800
C2—C13	1.495 (2)	C12—H12A	0.9800
C3—C4	1.391 (2)	C12—H12B	0.9800
C3—C8	1.396 (2)	C13—C18	1.391 (2)
C4—C5	1.393 (2)	C13—C14	1.395 (2)
C4—H4	0.9500	C14—C15	1.386 (2)
C5—C6	1.384 (2)	C14—H14	0.9500
C5—H5	0.9500	C15—C16	1.386 (2)
C6—C7	1.397 (2)	C15—H15	0.9500
C6—C9	1.508 (2)	C16—C17	1.383 (2)
C7—C8	1.383 (2)	C16—H16	0.9500
C7—H7	0.9500	C17—C18	1.390 (2)
C8—H8	0.9500	C17—H17	0.9500
C9—C10	1.532 (2)	C18—H18	0.9500
C9—H9B	0.9900		
			
N1—C1—N2	114.78 (12)	C11—C10—C9	113.76 (13)
N1—C1—S1	127.89 (11)	C11—C10—H10B	108.8
N2—C1—S1	117.32 (11)	C9—C10—H10B	108.8
C1—N1—C3	131.41 (13)	C11—C10—H10A	108.8
C1—N1—H1	114.3	C9—C10—H10A	108.8
C3—N1—H1	114.3	H10B—C10—H10A	107.7
C2—N2—C1	128.45 (13)	C12—C11—C10	112.35 (14)
C2—N2—H2	115.8	C12—C11—H11B	109.1
C1—N2—H2	115.8	C10—C11—H11B	109.1
O1—C2—N2	122.56 (14)	C12—C11—H11A	109.1
O1—C2—C13	121.20 (13)	C10—C11—H11A	109.1
N2—C2—C13	116.21 (13)	H11B—C11—H11A	107.9
C4—C3—C8	119.34 (13)	C11—C12—H12C	109.5
C4—C3—N1	125.23 (14)	C11—C12—H12A	109.5
C8—C3—N1	115.43 (13)	H12C—C12—H12A	109.5
C3—C4—C5	119.06 (14)	C11—C12—H12B	109.5
C3—C4—H4	120.5	H12C—C12—H12B	109.5
C5—C4—H4	120.5	H12A—C12—H12B	109.5
C6—C5—C4	122.41 (14)	C18—C13—C14	119.47 (14)
C6—C5—H5	118.8	C18—C13—C2	123.58 (13)
C4—C5—H5	118.8	C14—C13—C2	116.94 (14)
C5—C6—C7	117.70 (13)	C15—C14—C13	120.13 (15)
C5—C6—C9	120.87 (14)	C15—C14—H14	119.9
C7—C6—C9	121.34 (14)	C13—C14—H14	119.9
C8—C7—C6	120.92 (14)	C14—C15—C16	120.04 (15)
C8—C7—H7	119.5	C14—C15—H15	120.0
C6—C7—H7	119.5	C16—C15—H15	120.0
C7—C8—C3	120.55 (14)	C17—C16—C15	120.22 (15)
C7—C8—H8	119.7	C17—C16—H16	119.9
C3—C8—H8	119.7	C15—C16—H16	119.9
C6—C9—C10	111.53 (12)	C16—C17—C18	119.98 (15)
C6—C9—H9B	109.3	C16—C17—H17	120.0
C10—C9—H9B	109.3	C18—C17—H17	120.0
C6—C9—H9A	109.3	C17—C18—C13	120.14 (14)
C10—C9—H9A	109.3	C17—C18—H18	119.9
H9B—C9—H9A	108.0	C13—C18—H18	119.9
			
N2—C1—N1—C3	−178.89 (13)	N1—C3—C8—C7	179.89 (13)
S1—C1—N1—C3	2.0 (2)	C5—C6—C9—C10	83.10 (18)
N1—C1—N2—C2	−3.7 (2)	C7—C6—C9—C10	−93.57 (17)
S1—C1—N2—C2	175.53 (12)	C6—C9—C10—C11	−179.06 (14)
C1—N2—C2—O1	4.8 (2)	C9—C10—C11—C12	−177.71 (15)
C1—N2—C2—C13	−173.38 (13)	O1—C2—C13—C18	159.84 (15)
C1—N1—C3—C4	−9.2 (2)	N2—C2—C13—C18	−22.0 (2)
C1—N1—C3—C8	171.86 (14)	O1—C2—C13—C14	−20.9 (2)
C8—C3—C4—C5	−1.3 (2)	N2—C2—C13—C14	157.27 (14)
N1—C3—C4—C5	179.74 (14)	C18—C13—C14—C15	1.8 (2)
C3—C4—C5—C6	0.7 (2)	C2—C13—C14—C15	−177.51 (15)
C4—C5—C6—C7	0.5 (2)	C13—C14—C15—C16	−1.0 (3)
C4—C5—C6—C9	−176.28 (14)	C14—C15—C16—C17	−0.1 (3)
C5—C6—C7—C8	−1.0 (2)	C15—C16—C17—C18	0.2 (3)
C9—C6—C7—C8	175.77 (14)	C16—C17—C18—C13	0.6 (2)
C6—C7—C8—C3	0.3 (2)	C14—C13—C18—C17	−1.6 (2)
C4—C3—C8—C7	0.9 (2)	C2—C13—C18—C17	177.65 (14)

### Hydrogen-bond geometry (Å, °)

**Table:**

*D*—H···*A*	*D*—H	H···*A*	*D*···*A*	*D*—H···*A*
N1—H1···O1	0.88	1.88	2.630 (2)	142.
N2—H2···S1^i^	0.88	2.76	3.550 (2)	151.

Symmetry codes: (i) −*x*+1, −*y*, −*z*+1.

## References

[bb1] Allen, F. H. (2002). *Acta Cryst.* B**58**, 380–388. 10.1107/s0108768102003890 12037359

[bb2] Allen, F. H., Kennard, O., Watson, D. G., Brammer, L., Orpen, A. G. & Taylor, R. (1987). *J. Chem. Soc. Perkin Trans. 2* pp. S1–19.

[bb3] Altomare, A., Burla, M. C., Camalli, M., Cascarano, G. L., Giacovazzo, C., Guagliardi, A., Moliterni, A. G. G., Polidori, G. & Spagna, R. (1999). *J. Appl. Cryst.* **32**, 115–119.

[bb4] Johnson, C. K. (1976). *ORTEPII* Report ORNL-5138. Oak Ridge National Laboratory, Tennessee, USA.

[bb5] Kabuto, C., Akine, S., Nemoto, T. & Kwon, E. (2009). *J. Crystallogr. Soc. Jpn*, **51**, 218–224.

[bb6] Khawar Rauf, M., Bolte, M. & Badshah, A. (2009*a*). *Acta Cryst.* E**65**, o177. 10.1107/S1600536808042736 PMC2968087 21581633

[bb7] Khawar Rauf, M., Bolte, M. & Badshah, A. (2009*b*). *Acta Cryst.* E**65**, o240. 10.1107/S1600536809000063 PMC2968148 21581857

[bb8] Molecular Structure Corporation & Rigaku (2001). *CrystalClear.* MSC, The Woodlands, Texas, USA, and Rigaku Corporation, Tokyo, Japan.

[bb9] Molecular Structure Corporation & Rigaku (2004). *TEXSAN* MSC, The Woodlands, Texas, USA, and Rigaku Corporation, Tokyo, Japan.

[bb10] Sheldrick, G. M. (2008). *Acta Cryst.* A**64**, 112–122. 10.1107/S0108767307043930 18156677

